# Invariant natural killer T cells minimally influence gut microbiota composition in mice

**DOI:** 10.1080/19490976.2022.2104087

**Published:** 2022-07-31

**Authors:** Qiaochu Lin, Meggie Kuypers, Zhewei Liu, Julia K Copeland, Donny Chan, Susan J Robertson, Jean Kontogiannis, David S Guttman, E. Kate Banks, Dana J Philpott, Thierry Mallevaey

**Affiliations:** a Department of Immunology, University of Toronto, Toronto, ON, Canada; b Centre for the Analysis of Genome Evolution & Function, University of Toronto, Toronto, Ontario, Canada; c Division of Comparative Medicine, Faculty of Medicine, University of Toronto, Toronto, ON, Canada; d Department of Cell and Systems Biology, University of Toronto, Toronto, ON, Canada; e Department of Physiology, University of Toronto, Toronto, ON, Canada; f Institute of Biomaterials & Biomedical Engineering, University of Toronto, Toronto, ON, Canada

**Keywords:** iNKT cells, CD1d, α-galactosylceramide, microbiota, cross-fostering

## Abstract

Invariant Natural Killer T (iNKT) cells are unconventional T cells that respond to glycolipid antigens found in microbes in a CD1d-dependent manner. iNKT cells exert innate-like functions and produce copious amounts of cytokines, chemokines and cytotoxic molecules within only minutes of activation. As such, iNKT cells can fuel or dampen inflammation in a context-dependent manner. In addition, iNKT cells provide potent immunity against bacteria, viruses, parasites and fungi. Although microbiota-iNKT cell interactions are not well-characterized, mounting evidence suggests that microbiota colonization early in life impacts iNKT cell homeostasis and functions in disease. In this study, we showed that CD1d^−/−^ and Vα14 Tg mice, which lack and have increased numbers of iNKT cells, respectively, had no significant alterations in gut microbiota composition compared to their littermate controls. Furthermore, specific iNKT cell activation by glycolipid antigens only resulted in a transient and minimal shift in microbiota composition when compared to the natural drift found in our colony. Our findings demonstrate that iNKT cells have little to no influence in regulating commensal bacteria at steady state.

**Abbreviations:** iNKT: invariant Natural Killer T cell; αGC: α-galactosylceramide

## Introduction

The mammalian immune system is equipped with diverse populations of unconventional T cells that respond to antigens otherwise invisible to conventional CD4^+^ and CD8^+^ T cells. These unconventional T cells include γδ T cells, mucosa-associated invariant T (MAIT) cells and invariant natural killer T (iNKT) cells.^1^ iNKT cells respond to glycolipid antigens, such as α-galactosylceramide (αGC), presented by the non-polymorphic major histocompatibility complex (MHC) class Ib molecule CD1d^2^. iNKT cells have a restricted T cell receptor (TCR) repertoire characterized by a canonical and invariant TCRα chain (Vα14-Jα18 in mice and Vα24-Jα18 in humans) paired with a limited set of TCRβ chains. iNKT cells produce copious amounts of a large array of cytokines (e.g. IFN-γ, TNF-α, IL-4, IL-17A), chemokines and cytotoxic molecules within minutes following activation, which can influence other innate and adaptive immune cells.^1,2^ As such, iNKT cells participate in anti-infectious and anti-tumor immunity and can positively or negatively regulate autoimmune and inflammatory responses.

iNKT cells recognize α-galacturonosylceramides from species of *Sphingomonas* and *Ehrlichia*,^3,4^ α-glucosyldiacylglecerols from *Borrelia burgdorferi* and *Streptococcus pneumoniae*,^5,6^ as well as other microbe-derived lipid-based antigens.^7^ Commensal bacteria such as *Bacteroides fragilis* also express glycolipids capable of activating iNKT cells.^8–10^ Microbe-induced pro-inflammatory cytokines, such as IL-12 and/or IL-18 can also activate iNKT cells, which seems to be a prominent activation pathway during bacterial and viral infection.^7,11–13^ Other microbial molecules or their by-products can regulate iNKT cell activation. These include short chain fatty acids, tryptophan metabolites, bile acids, and oxazoles.^7^ iNKT cells participate in bacterial^3,6,14–16^ and viral^17–22^ clearance, which can be achieved through the recruitment and/or activation of macrophages, neutrophils and B cells.

The intestinal microbiota regulates the development, maturation and function of a variety of immune cells.^23,24^ Germ-free (GF) mice, which are devoid of commensal microflora, have hypotrophic and disorganized Peyer’s patches as well as reduced IgA-producing plasma cells and CD4^+^ T cells in the intestinal lamina propria.^25^ Colonization of GF mice by individual microbial species or defined consortia can restore defects in specific mucosal CD4^+^ T cell subsets.^23^ For example, colonization of mice with either segmented filamentous bacteria (SFB) or the protozoa *Tritrichomonas* stimulates the development of intestinal T_H_17 cells and/or T_H_1 cells,^26–28^ while the colonization with consortia of *Clostridium* species induces the development of regulatory T cells.^29–31^ In addition, feral and pet store mice, which have a broader environmental exposure, have increased prevalence of circulating effector/memory T cells.^32–35^ iNKT cell homeostasis and functions are altered in GF mice^36,37^ and early-life colonization by the microbiota limits their accumulation at mucosal surfaces and regulates their pro-inflammatory role in induced models of asthma and colitis.^9,36^

Whether specific T cell populations, including unconventional T cells, or their by-products influence the composition of the intestinal microbiota remains unclear.^7^ Specifically, several studies have reported alterations of the gut microbiota composition in iNKT cell-deficient mice.^38–42^ In this study, we have analyzed the microbiota of CD1d^−/−^ mice, which lack iNKT cells, and Vα14 transgenic (Tg) mice, which have increased iNKT cell numbers. We found that these mice had no consistent alteration in their microbiota composition compared to their respective wild-type littermate controls. Furthermore, specific iNKT cell activation by glycolipid ligands such as αGC led to a moderate and transient shift in composition. This shift was not consistently found between mouse colonies and was minimal compared to the natural drift that occurred in our mouse colony over time. In addition, we found that maternal transmission and caging had greater impacts on microbiota composition than the absence, overrepresentation, or activation of iNKT cells. Together, these data suggest that iNKT cells have little to no influence on the composition of the intestinal microbiota in mice.

## Results

### Cross-fostering of CD1d-deficient mice impacts microbiota composition

**Figure 1.**
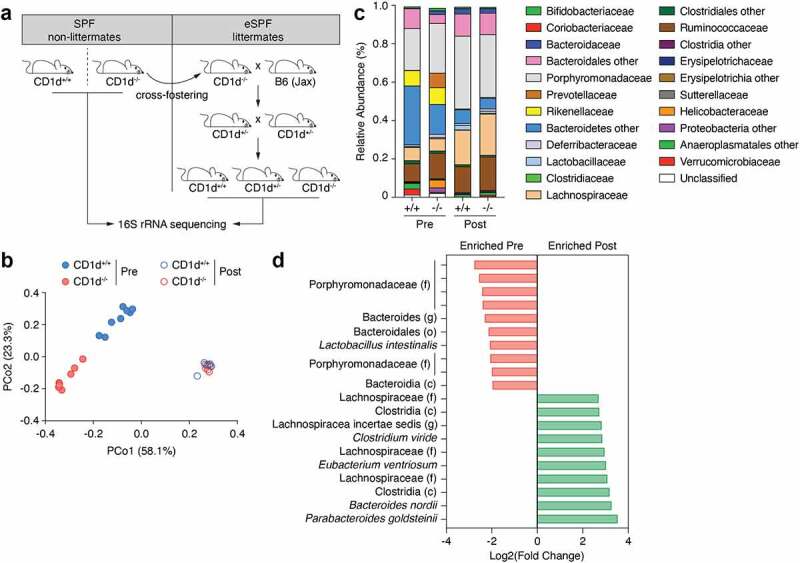
**Cross-fostering of CD1d^−/−^ mice impacts microbiota composition**. (a) Schematic of cross-fostering, breeding and housing. (b) 16S bacterial rRNA sequencing was used to define the microbiota profiles of CD1d^+/+^ and CD1d^−/−^ non-littermate mice prior to cross-fostering (Pre) and CD1d^+/+^ and CD1d^−/−^ littermate mice post-cross-fostering (Post) (n = 6 to 8 mice per group). These groups were separated by principal coordinates PCo1 and PCo2, collectively explaining 83.4% of the total similarity between samples, based on Bray-Curtis distances. (c) Relative abundance (family level) varied between CD1d^+/+^ and CD1d^−/−^ mice pre-cross-fostering, as well as between mice pre- and post-cross-fostering. (d) MetagenomeSeq analysis shows the top 10 most enriched OTUs in CD1d^−/−^ mice pre- and post-cross-fostering. Log2 fold-change values are shown.

We previously reported that CD1d-deficient mice harbored a proinflammatory microbiota that exacerbated colitis induced by dextran sodium sulfate (DSS).^43^ This microbiota was characterized by the presence of SFB and the increased abundance of certain phyla including Proteobacteria, Deferribacteres, as well as *Prevotella* and *Mucispirillum* spp. As the microbiota composition was compared to that of non-littermate wild type (WT) B6 mice, it remained unclear whether these differences were a consequence of adjacent breeding or driven by the lack of CD1d and the absence of iNKT cells. We first attempted to rid CD1d-deficient mice of certain microbes that are known to influence the immune landscape and/or fuel inflammation, such as *Helicobacter hepaticus*,^44^ Tritrichomonas species^27,28^ and the mouse norovirus (MNV).^45^ To achieve this, we cross-fostered CD1d^−/−^ newborn pups within 24 h of birth and subsequently housed them in a designated suite with enhanced specific pathogen-free (eSPF) status.^46^ CD1d heterozygous eSPF breeding pairs were established for colony maintenance and subsequent experiments (Figure 1a). PCR testing for common mouse pathogens revealed that cross-fostered mice were devoid of Helicobacter, MNV and Tritrichomonas (Table 1 and not shown).

**Table 1.** Mice were tested for the presence of mouse norovirus (MNV) and several Helicobacter species before (Pre) and after (Post) cross-fostering.

	Pre	Post
MNV	+	-
*H. bills*	-	-
*H. ganmani*	-	-
*H. hepaticus*	-	-
*H. mastomyrinus*	+	-
*H. rodentium*	-	-
*H. typhlonius*	+	-

We next analyzed the colon microbial communities from eSPF CD1d^+/+^ and CD1d^−/−^ littermate mice (post-cross-fostering [Post]) and compared to that of SPF CD1d^+/+^ and CD1d^−/−^ non-littermate mice (pre-cross-fostering [Pre]) via 16S rRNA sequencing (Figure 1b). The principal coordinates analysis (PCoA) generated through Bray-Curtis dissimilarity revealed that pre- and post-cross-fostering mice segregated along PCo1 (58.1% of the variance), while pre-cross-fostering CD1d^+/+^ and CD1d^−/−^ mice segregated along PCo2 (23.3% of the variance). Although post-cross-fostering eSPF CD1d^+/+^ and CD1d^−/−^ littermate mice appeared to cluster together, they were significantly different from both CD1d^+/+^ and CD1d^−/−^ non-littermate SPF mice, indicating that the cross-fostering procedure altered microbiota composition. Relative abundance plots revealed that the microbiota from cross-fostered (Post) mice had reduced abundance of Erysipelotrichaceae, Rikinellaceae and Bacteroidetes, and was almost completely devoid of Prevotellaceae, Helicobacter-iaceae and Proteobacteria (Figure 1c). On the other hand, cross-fostered mice had increased abundance of Lachnospiraceae (Figure 1c). The relative abundance of operational taxonomic units (OTUs) in the pre- and post-cross-fostering groups were compared using a combination of MetagenomeSeq and DESeq2 analyses.^47^ These combined methods revealed that 85 OTUs were significantly different between pre- and post-cross-fostering mice (Supplementary Table 1). The top 10 OTUs enriched in pre- and post-cross-fostering are depicted in Figure 1d. Specifically, 17 OTUs were enriched in pre-cross-fostering samples including several members of Porphyromonadaceae as well as *Lactobacillus intestinalis*. On the other hand, 68 OTUs, including several species of Lachnospiraceae and Clostridia, were significantly enriched in post-cross-fostering mice. In sum, these results demonstrate that cross-fostering of newborn CD1d^−/−^ mice changed the composition of the intestinal microbiota compared to their birth mothers.

### CD1d deficiency does not affect microbiota composition in littermate mice

**Figure 2.**
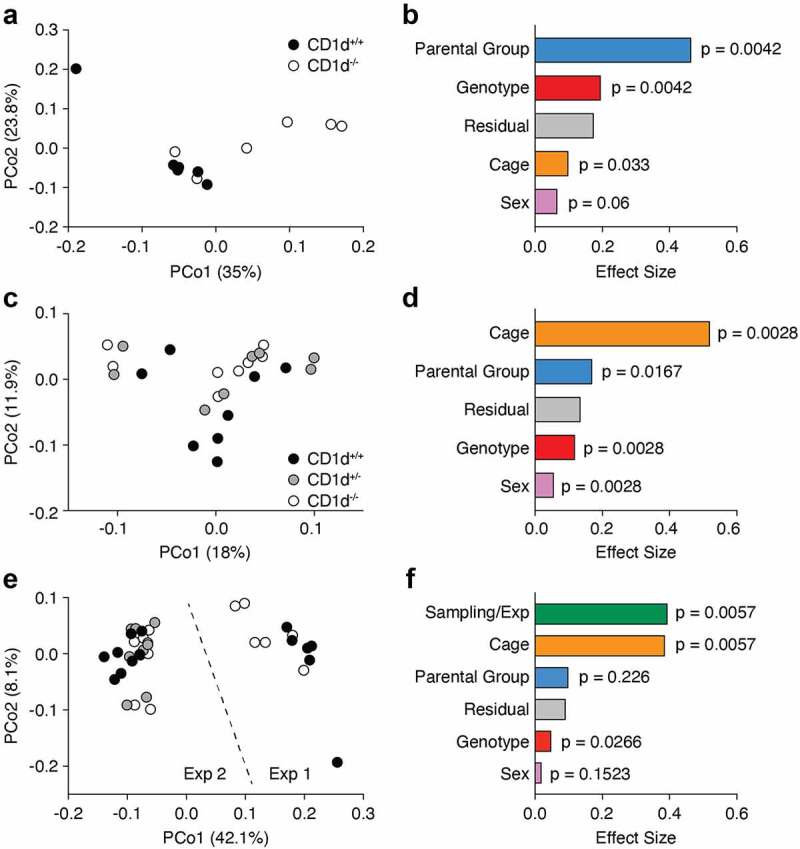
**CD1d-deficiency and loss of iNKT cells does not alter microbiota composition**. (a, b) 16S bacterial rRNA sequencing was used to define the microbiota profiles of CD1d^+/+^ and CD1d^−/−^ littermate mice (n = 6 mice per group). These groups were separated by principal coordinates PCo1 and PCo2, collectively explaining 58.8% of the total similarity between samples, based on Bray-Curtis distances (a). Permutational multivariate analysis of the variance using Adonis (b). Data shows R^2^ (effect size) and adjusted *p* values for genotype, sex, caging and parental group. (c, d) 16S bacterial rRNA sequencing was used to define the microbiota profiles of CD1d^+/+^, CD1d^+/−^ and CD1d^−/−^ littermate mice (n = 8 mice per group) obtained from 2 consecutive litters from heterozygous harem breeding (1 male + 3 females). These groups were separated by principal coordinates PCo1 and PCo2, collectively explaining 29.9% of the total similarity between samples, based on Bray-Curtis distances (c). Permutational multivariate analysis of the variance using Adonis (d). Data shows R^2^ (effect size) and adjusted *p* values for genotype, sex, caging and parental group. (e, f) Combined Bray-Curtis PCoA of the two experiments (e) and Adonis (f) shows R^2^ (effect size) and adjusted *p* values for genotype, sex, caging, parental group and sampling/experiment.

We next focused on the comparison between CD1d^+/+^ and CD1d^−/−^ littermate mice that were eSPF status after cross-fostering (experiment 1). Of note, mice were caged according to sex and genotype after weaning. The PCoA plot generated from Bray-Curtis dissimilarity showed that the bacterial communities found in the proximal colon fecal matter of CD1d^+/+^ and CD1d^−/−^ littermate mice were significantly different (*p* < 0.0042) (Figure 2a,b), whereas there was no statistical difference found in fecal pellets (not shown). However, permutational multivariate analysis of the variance (PERMANOVA) using Adonis revealed that genotype only accounted for about 20% of the observed variance (effect size or R^2^ = 0.195), (Figure 2b). On the other hand, parental group accounted for most of the observed variance (R^2^ = 0.464). Combined MetagenomeSeq and DESeq2 analyses showed that no OTU was significantly different between genotypes (not shown). This suggested that parental vertical transmission had a greater effect on microbiota composition than the lack of CD1d and the absence of iNKT cells.

To minimize the variability between dams and mitigate parental transmission, we set up a CD1d heterozygous breeding harem (1 male + 3 dams). We separated the pregnant dams from the male before delivery and we sequenced the proximal colon microbiota of 2 consecutive litters from these 3 dams (experiment 2). Although the PCoA showed no obvious segregation between CD1d^+/+^, CD1d^+/−^ and CD1d^−/−^ mice, the genotype significantly (Adonis *p* < 0.0028) impacted microbiota composition (Figure 2c, d). However, the genotype only accounted for ~12% of the variance. In these settings, parental group and caging accounted for 17% and 52% of the variance, respectively (Figure 2d). Again, no OTU was significantly differentially represented between genotypes (not shown). When the two experiments were combined, the contribution of the genotype became even less apparent (R^2^ = 0.048, *p* < 0.0266) Figure 2(e,f) and no OTU was found to be significantly different between genotypes (not shown). Parental group and caging accounted for ~10% and 40% of the variance, respectively (figure 2f). Sampling, or experiment, also accounted for 40% of the observed variance. When comparing all samples from experiments 1 and 2, combined MetagenomeSeq and DESeq2 analyses revealed that only 2 OTUs of the *Clostridium IV* and *Oscillibacter* genera (Ruminococcaceae), were significantly enriched in experiment 1 compared to experiment 2 (not shown).

As it has been suggested that genes can have context-dependent effects on microbiota composition,^48^ we next compared microbial communities from CD1d-sufficient and CD1d-deficient littermate mice housed in a SPF suite (not cross-fostered). Due to low availability of samples from CD1d^+/+^ mice, we used CD1d^+/−^ mice, which have the same iNKT cell frequency, absolute number, and effector subset distribution as CD1d^+/+^ mice in all tissues tested (thymus, spleen, liver and mesenteric lymph nodes) (**Supplementary Fig. 1A, B** and not shown). The PCoA generated from Bray Curtis distances revealed no statistical difference in microbiota composition between CD1d^+/−^ and CD1d^−/−^ littermate mice, both in the terminal ileum and proximal colon (**Supplementary Fig. 1C, D**). Taken together, these results demonstrated that CD1d deficiency, and the consequential lack of iNKT cells, does not measurably and reproducibly impact microbiota composition beyond the “natural drift” observed in our mouse colony, which was primarily driven by maternal vertical transmission and the random selection of breeders for colony maintenance.

### Increased iNKT cell prevalence does not impact microbiota composition

**Figure 3.**
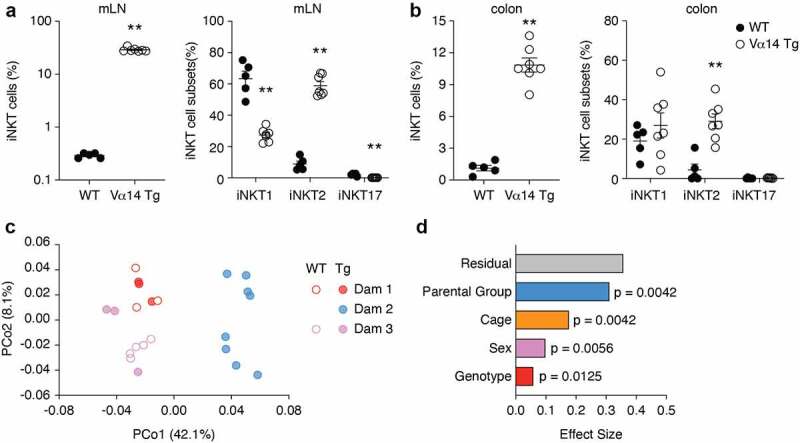
**Increased iNKT cells prevalence in V**α**14 Tg mice does not impact microbiota composition**. (a, b) Frequency of TCRβ^+^ PBS57-CD1d tetramer-positive iNKT cells out of live CD19^−^ lymphocytes and subset distribution of iNKT cell subsets in the mesenteric lymph nodes (mLN, A) and colon (b) of WT and Vα14 Tg mice. Data shows individual and mean values ± s.e.m. (n = 3 to 4 mice per group). **p < 0.01  , Mann-Whitney. (c) 16S bacterial rRNA sequencing was used to define the microbiota profiles of WT and Vα14 Tg littermate mice. These groups were separated by principal coordinates PCo1 and PCo2, collectively explaining 50.2% of the total similarity between samples, based on Bray-Curtis distances. 8 WT and 14 Vα14 Tg mice from 3 litters were analyzed. (d) Permutational multivariate analysis of the variance using Adonis. Data shows R^2^ (effect size) and adjusted *p* values for genotype, sex, caging and parental group.

Mice that overexpress the rearranged Vα14-Jα18 iNKT TCRα chain (Vα14 Tg) have increased prevalence of iNKT cells^49,50^ Figure 3(a,b). In addition, iNKT cell effector differentiation is severely biased toward the iNKT2 subset in these Vα14 Tg mice^50,51^ Figure 3(a,b), which predominantly produces IL-4.^52^ To assess whether this impacted microbiota composition, we sequenced the proximal colon microbiota from Vα14 Tg and WT littermate mice from 3 litters resulting from a harem (1 male + 3 dams). The PCoA generated from Bray Curtis distances revealed no obvious segregation based on genotype, which only accounted for 6% of the variance Figure 3(c,d). Mice from the second dam clearly segregated from the other two groups along PCo1, and parental group accounted for about 30% of the variance (*p* < 0.0042). Sex and cage accounted for 10% and 17% of the variance, respectively (Figure 3d). No OTU was significantly different between Vα14 Tg and WT mice. These results demonstrate that the increased prevalence of iNKT cells in Vα14 Tg mice has no measurable impact on gut microbiota composition.

### iNKT cell activation minimally and transiently impacts microbiota composition

**Figure 4.**
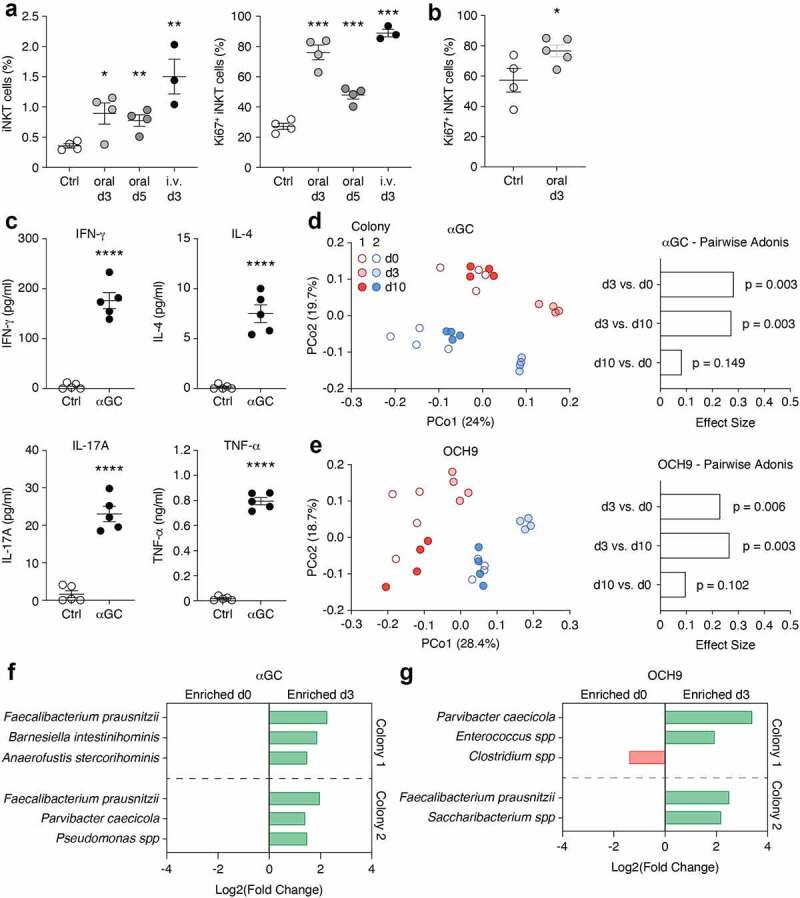
**iNKT cell activation transiently affects microbiota composition**. (a) C57BL/6 mice were administered 2 μg αGC or vehicle control (Ctrl) orally, or 0.5 μg αGC intravenously (i.v.) and their mLN were analyzed at d3 and d5 (for oral) and d3 (i.v.). (b) C57BL/6 mice were administered 2 μg αGC or vehicle control (Ctrl) and their colons were analyzed at d3. The data shows the frequency of iNKT cells out of live CD19^−^ lymphocytes (a) as well as the frequency of Ki67^+^ iNKT cells (a, b). (c) C57BL/6 mice were administered 2 μg αGC or vehicle control (Ctrl). At d3, colon biopsy punches were cultured for 48 h and cytokines production was assessed using a multiplex cytokine array. Data shows individual and mean values ± s.e.m. (n = 3 to 5 mice per group). *p < 0.05, **p < 0.01, *** *p* < 0.001, **** *p* < 0.0001, Unpaired Student *t* test (a-c). (d, e) 16S bacterial rRNA sequencing was used to define the microbiota profiles of mice before (d0). 3 (d3) and 10 days (d10) after the oral gavage of αGC (d) or OCH9 (e). These groups were separated by principal coordinates PCo1 and PCo2, collectively explaining 43.7% (d) and 47.1% (e) of the total similarity between samples, based on Bray-Curtis distances (left panels). Pairwise Adonis comparison of microbiota composition at d0, d3 and d10 following αGC (d) or OCH9 (e) administration is shown (right panels). (f, g) MetagenomeSeq analysis shows differentially abundant OTUs before (d0) and after (d3) oral administration of αGC (f) or OCH9 (g).

We next selectively activated iNKT cells in WT mice through the gavage of either αGC or the analog OCH9, which has been shown to induce a T_H_2-biased cytokine response.^53^ We found that oral gavage of αGC induced proliferation of iNKT cells in the spleen, mesenteric lymph nodes and colon lamina propria, with a peak at 72 h (d3), as assessed by iNKT cell frequency and Ki67 expression Figure 4(a,b) and **Supplementary Fig 2A**).^38,54–56^ In addition, colons from αGC-gavaged mice produced significantly higher amounts of IFN-γ, IL-4, IL-17A, TNF-α, IL-1β, IL-2 and IL-12p70, but not IL-13, GM-CSF, M-CSF and RANTES (Figure 4c**, Supplementary Fig. 2B** and not shown). Elevated amount of IL-5 and MCP-1, known to recruit eosinophils and monocytes, respectively, were also produced by the colons of αGC-treated mice (**Supplementary Fig. 2B**). We found no difference in cytokine and chemokine production in the sera of αGC-treated mice compared to control mice (not shown). Together, these data show that oral administration of αGC leads to iNKT cell activation and the local production of cytokines and chemokines.

With this in mind, we sequenced the fecal pellet microbiota from mice before, as well as 3 and 10 days following the gavage of αGC or OCH9. We used mice born to two B6 dams from our in-house colony (colony 1) as well as mice born to two dams recently acquired from the Jackson Laboratories (colony 2). PCoA analyses from the Bray Curtis distances revealed that the 2 colonies segregated mainly along PCo2 for αGC and PCo1 for OCH9 Figure 4(c,d). Both αGC and OCH9 induced a significant shift in microbiota composition 3 days post-gavage Figure 4(c,d). By day 10, the microbiota composition was not significantly different than prior to gavage. This suggested that iNKT cells activation by αGC or OCH9 induced a transient shift in microbiota composition. However, only a few OTUs were significantly differentially abundant at day 3 and these differences were not consistent between colonies and antigens Figure 4(e,f). Only *Faecalibacter prausnitzii* was consistently enriched at day 3 following activation. Finally, to assess whether repeated iNKT cell stimulation had a greater impact on microbiota composition, we treated mice with αGC orally once per week for 3 consecutive weeks (**Supplementary Fig. 2C**). PCoA analyses from the Bray Curtis distances revealed that αGC-treated and control mice clustered together by day 21, revealing a minimal shift in microbiota composition (**Supplementary Fig. 2D, E**). Combined MetagenomeSeq and DESeq2 analyses revealed no difference in OTUs in αGC-treated mice between day 0 and day 21 (not shown). Together these data suggest that iNKT cell activation by glycolipid antigens only minimally and transiently impacts gut microbiota composition.

### The microbiota composition in wild-type mice shifts over time

**Figure 5.**
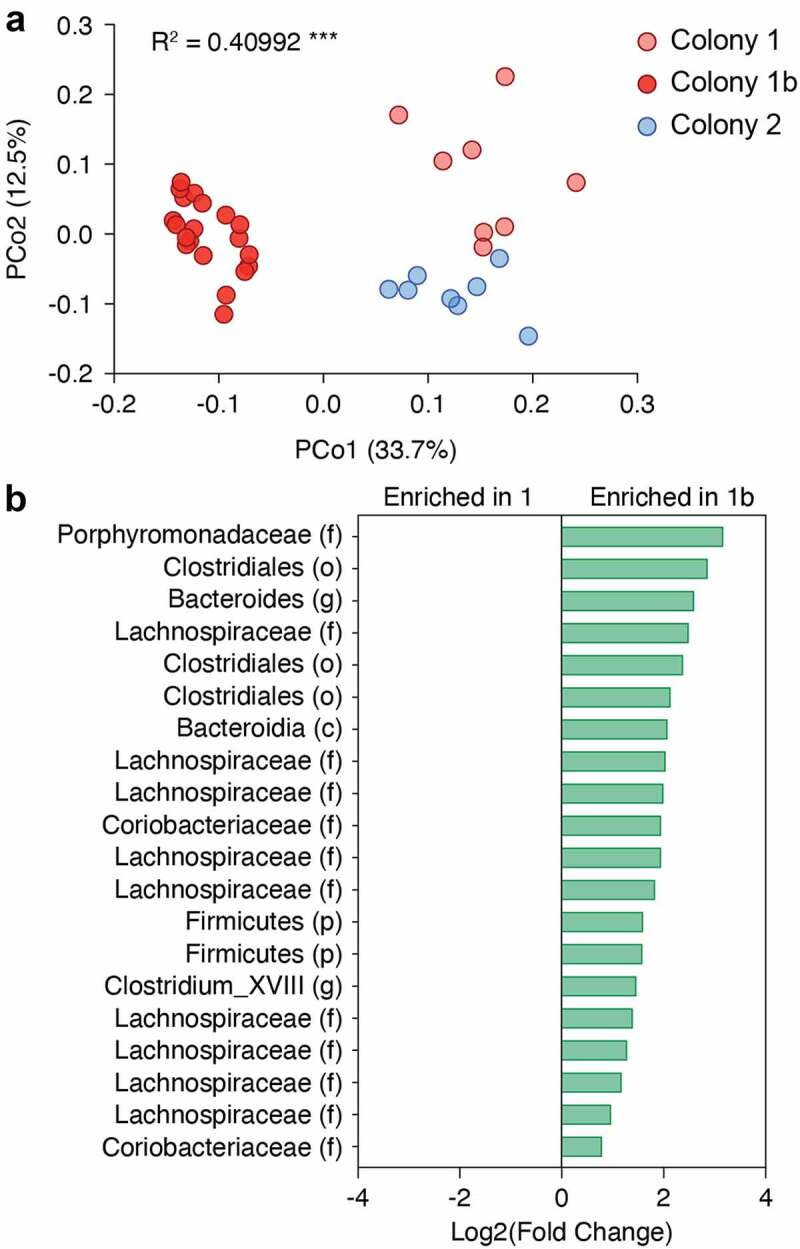
**The composition of the microbiota in C57BL/6 mice shifts over time**. (a) 16S bacterial rRNA sequencing was used to define the microbiota profiles of C57BL/6 mice sampled from the same colony one year apart (colonies 1, n = 8, and 1b, n = 20) and from a separate colony (colony 2, n = 8). These groups were separated by principal coordinates PCo1 and PCo2, collectively explaining 46.2% of the total similarity between samples, based on Bray-Curtis distances. (b) MetagenomeSeq analysis shows the top 10 most enriched OTUs in colony 1 and the 5 OTUs enriched in colony 1b. Log2 fold-change values are shown.

To assess whether the composition of the microbiota changed over time in our in-house C57BL/6 colony (colony 1 used above), we sampled mice from the same colony again approximately 12 months later (colony 1b). We also included mice from colony 2 described above in our comparison. PCoA analyses from the Bray Curtis distances revealed that colonies 1 and 1b segregated mainly along PCo1, which accounted for 33.7% of the variance, while colonies 1 and 2 separated along PCo2, which accounted for 12.5% of the variance (Figure 5a). PERMANOVA using Adonis revealed that the 3 colonies had significantly distinct microbiotas (R^2^ = 0.40992, p < 0.001). Through combined MetagenomeSeq and DESEq2 analyses, we found that 20 OTUs were significantly enriched in colony 1b, including several members of the Clostridiales order and the Lachnospiraceae and Coriobacteriaceae families (Figure 5b). These results indicated that the composition of the microbiota in our in-house colony shifted significantly over time.

## Discussion

In this study, we have shown that mice that lack iNKT cells (CD1d^−/−^ mice) and mice that have increased iNKT cell numbers (Vα14 Tg mice) had no significant alterations in the composition of their intestinal microbiota relative to their respective littermate controls. Specific iNKT cell activation by 2 distinct glycolipid antigens (αGC and OCH9) led to a transient shift in microbiota composition, mainly characterized by increased abundance of *F. prausnitzii*. Together, our work suggests that iNKT cells have little influence on the composition of the intestinal microbiota at steady state, and that their activation only transiently shifts microbiota composition.

Differences in microbiota composition have been reported in CD1d^−/−^ or Jα18^−/−^ mice, the latter specifically lacking iNKT cells due to the inability to generate the canonical Vα14-Jα18 TCRα chain, compared to wild-type mice.^38–42^ The discrepancy between these studies and ours most likely lies in the experimental design and methods of analysis used. In some of the published studies, it is unclear whether iNKT cell-deficient and sufficient littermate mice were used, in other words, mice that were the product of heterozygous breeding. Comparing groups of mice that are bred separately, or sourced from different facilities (e.g. wild-type mice purchased from outside vendors), has clear limitations and does not provide conclusive evidence that the genotype studied drives the observed difference in microbiota composition.^57,58^ Although co-housing of iNKT cell-deficient and -sufficient mice has been used in efforts to normalize the microbiota for several weeks prior to sampling, such methods have been proven ineffective at normalizing microbiota composition, especially for mucosa-associated microbes.^59,60^ Here, we have used littermate mice and accounted for other confounding factors in our analyses, such as sex, caging and parental transmission. Our results clearly demonstrate that iNKT cells or their specific activation only minimally influence microbiota composition. In fact, in experimental settings using harem breeding (and sire removal prior to delivery), maternal transmission and caging invariably had a greater impact on microbiota composition than genotype.

Many factors such as diet, lifestyle, metabolism, and infection history regulate the microbiota. However, the relative contribution of host genetics on microbiota composition remains unclear. Although genetic manipulation in otherwise inbred mice offers the possibility to study how specific genes and their products impact microbiota composition, these studies have failed to provide unifying findings. This is the case of studies of mammalian innate receptors involved in the recognition of microbe-associated molecular patterns (MAMPs). Such molecules seem like ideal candidates, and initial studies indeed reported that deficiencies in toll-like receptor (TLR) 5 or the adaptor protein MyD88 changed the microbiota composition.^61,62^ However, a subsequent study using littermate mice revealed that deficiency in MyD88 or several TLRs did not impact microbiota composition, richness, or diversity.^57^ Similarly, mice lacking the Nod-like receptor NLRP6 or inflammasome components such as ASC were shown to have fecal dysbiosis,^63,64^ but multiple subsequent studies have failed to reproduce these data.^65–67^ It has been argued that genetic influence on the microbiota could be context-specific (e.g. particular microbiota composition in a given animal facility) and that microbiota studies in gene-targeted mice should be conducted with several microbiota environments, such as through fecal microbiota transfer.^48,68^ However, we found no difference in microbiota composition between CD1d-deficient and sufficient littermate mice before and after cross-fostering, housed in SPF and eSPF conditions, respectively. In humans, multiple studies of Dutch, Flemish and Israeli populations found that host genetics plays a minor role in determining microbiota composition.^69–71^ A key finding from these studies is that confounding factors should be considered when analyzing human data^72^ and when designing preclinical mouse studies, such as the systematic use of littermate-controlled experiments.^57–59,68^

Cross-fostering is a simple and effective method for generating mice with a desired gut microbiota. Multiple studies have demonstrated that newborn pups fostered by dams with a different microbiota develop the same microbiota and associated phenotypes as their nursing mother rather than their birth mother.^46,64,73,74^ In line with this, cross-fostering of our SPF CD1d-deficient mice with surrogate mothers in barrier conditions effectively eliminated several microbes that are known to alter the microbiota and immune landscape,^27,28,44,45,75–78^ and engrafted the mice with a new conventional microbiota from which we could then track how it is selectively shaped by CD1d and/or iNKT cells over time. However, a question that may arise from experiments that study the impact of host genetics on the microbiota is how long should the microbiota be monitored to determine whether a genotype effect becomes biologically relevant? In this study, we examined the microbiota composition of littermate mice at 7 to 8 weeks of age to match with previous studies that reported the age of the mice used.^39–42^ While we found that the effect of genotype on microbiota was indeed statistically significant, it was minimal compared with other non-genetic factors. Consistent with our finding, a recent study by Viennois et *al*. demonstrated that the influence of peptide transporter 1 (PepT1) deficiency on the microbiota is minimal between F1 PepT1^+/+^ and PepT1^−/−^ littermates.^79^ The effect of PepT1 gradually emerged along with its associated phenotypes after several generations of separate homozygous breeding of PepT1^+/+^ and PepT1^−/−^ mice from the initial intercross, which suggests that certain gene mutations may take longer to exert their effect on the microbiota.^79^ However, a limitation of such experimental design is that it becomes unclear whether the ecological drift in the microbiota is driven by the genotype or randomly introduced by adjacent breeding. Our work showed that the microbiota of our WT mouse colony can shift over time despite consistent housing conditions and absence of genetic manipulation. This is likely due to random breeder selection, wherein certain inherent microbiota differences within and between breeding pairs are selectively propagated through their progeny, ultimately leading to a change in the microbial landscape of the entire colony.^80^ Therefore, any microbiota changes observed in a gene-targeted mouse line that is bred and maintained in isolation must be interpreted with care in order to determine the true effect of the host genetics. Nevertheless, we cannot exclude the possibility that the influence of CD1d and/or iNKT cells may increase with age, and hence the microbiota of older CD1d littermate mice may warrant further examination in subsequent studies. In addition, it is also possible that iNKT cell deficiency could impact microbiota resilience (i.e. the ability to recover from perturbations), which has been associated with better health.^81^ In line with this, Nod2-/- mice have altered and/or delayed microbiota recovery after antibiotic treatment, which impacts colitis susceptibility.^82,83^

iNKT cell activation by the specific glycolipid ligands αGC and OCH9 led to a transient shift in microbiota composition, characterized by an increased abundance of *F. prausnitzii*. Interestingly, a low proportion of *F. prausnitzii* was associated with recurrence of Crohn’s disease in patients following surgical resection.^84^ In addition, *F. prausnitzii* was shown to exert anti-inflammatory functions in experimental colitis in mice.^84^ This could explain the beneficial effects of αGC and OCH9 treatment during dextran sodium sulfate (DSS)-induced colitis.^85,86^ However, we have not been able to reproduce these findings, and our previous findings suggested that the outcome of OCH9 treatment could be modulated by the intestinal microbiota.^43^

In sum, we found that mice that are genetically deficient in iNKT cells or mice that have increased frequency of these cells have no alteration in their microbiota composition at steady state. In addition, their specific activation in wild-type mice leads to a moderate and transient shift in microbiota composition. Together, our findings suggest that iNKT cells have little to no influence on the composition of intestinal commensal bacteria in mice.

## Materials and methods

### Mice and reagents

Mice were used between 6–8 weeks of age, unless otherwise specified. C57BL/6 (B6) mice were purchased from Jackson Laboratories. CD1d^−/−^ mice were generated and generously provided by Dr. Chyung-Ru Wang (Northwestern University, USA).^87^ Vα14 Tg mice were a generous gift from Dr. Albert Bendelac (University of Chicago).^49^ All strains were bred at the Division of Comparative Medicine, University of Toronto animal facility under barrier conditions in separate designated areas, with either specific-pathogen free (SPF) or enhanced (e) SPF status. The mice used in this study were the product of in-house heterozygous breeding pairs or triads, unless specified. All animal procedures were approved by the Faculty of Medicine and Pharmacy Animal Care Committee at the University of Toronto (Animal use protocols 20010135, 20010715, 20011113, 20011656). α-galactosylceramide (KRN7000, αGC) was purchased from Diagnocine. Antibodies used were purchased from ThermoFisher Scientific, Biolegend or BD Biosciences. OCH9, as well as PBS57-loaded and unloaded biotinylated CD1d monomers were obtained from the NIH Tetramer Core Facility. CD1d monomers were tetramerized by addition of fluorochrome-conjugated streptavidin.

### Cross-fostering procedure & husbandry

Outbred CD-1 female mice were used as surrogate mothers and housed under barrier conditions within a designated quarantine suite within the Division of Comparative Medicine. CD1d^−/−^ mice were transferred to quarantine and timed pregnancies were set. Pregnant females were transferred into clean autoclaved cages 2 days prior to delivery. Within 24 h of birth, pups were placed on warm and wet PREempt wipes on a heating pad and under a heating lamp in a biosafety cabinet for 2 min. Pups were then placed with surrogate outbred female mice that had littered within the past 5 days (all outbred pups but one were removed). Fecal pellets were collected from cages housing cross-fostered pups for PCR testing to confirm the elimination of Helicobacter spp., MNV and Tritrichomonas. These CD1d^−/−^ mice were then bred with C57BL/6 mice to generate CD1d^+/−^ mice. This colony was maintained as CD1d^+/−^ x CD1d^+/−^ breeding pairs housed in barrier conditions in the suite designated with eSPF status. After breeding CD1d^+/−^ mice together for 5 generations, CD1d^+/+^, CD1d^+/−^ and CD1d^−/−^ littermate mice were used for our studies.

### 16S rRNA sequencing and OTU assembly

The V4 hypervariable region of the 16S rRNA gene was amplified using uniquely barcoded 515 F (forward) and 806 R (reverse) sequencing primers to allow for multiplexing.^88^ Amplification reactions were performed using 12.5 μl of KAPA2G Robust HotStart ReadyMix (KAPA Biosystems), 1.5 μl of 10 μM forward and reverse primers, 7.5 μl of sterile water and 2 μl of DNA. The V4 region was amplified by cycling the reaction at 95**°**C for 3 minutes, 22x cycles of 95**°**C for 15 seconds, 50**°**C for 15 seconds and 72**°**C for 15 seconds, followed by a 5-minute 72**°**C extension. All amplification reactions were done in triplicate to reduce amplification bias, pooled, and checked on a 1% agarose TBE gel. Pooled triplicates were quantified using PicoGreen and combined by even concentrations. The library was then purified using Ampure XP beads and loaded on to the Illumina MiSeq for sequencing, according to manufacturer instructions (Illumina, San Diego, CA). Sequencing was performed using the V2 (150bp x 2) chemistry. A single-species (*Pseudomonas aeruginosa* DNA), a mock community (Zymo Microbial Community DNA Standard) and a template-free negative control were sequenced.

The UNOISE pipeline, available through USEARCH v11.0.667 and vsearch v2.10.4, was used for sequence analysis.^89,90^ The last base was removed from all sequences using cutadapt v.1.18. Sequences were assembled and quality trimmed using – fastq_mergepairs with a – fastq_trunctail set at 2, a – fastq_minqual set at 3, a -fastq_maxdiffs set at 5, a -fastq_pctid set at 90, and minimum and maximum assemble lengths set at 243 and 263 (± 10 from the mean) base pairs. Assembled sequences were quality filtered using – fastq_filter with a – fastq_maxee set at 1.0. The trimmed data was then processed following the UNOISE pipeline. Sequences were first de-replicated and sorted to remove singletons, then denoised and chimeras were removed using the unoise3 command. Assembled sequences were mapped back to the chimera-free denoised zOTUs at 99% identity, allowing for a 1% PCR error-rate. Taxonomy assignment was executed using SINTAX, available through USEARCH, and the UNOISE compatible Ribosomal Database Project (RDP) database version 16, with a minimum confidence cutoff of 0.8. OTU sequences were aligned using align_seqs.py v.1.9.1 through QIIME1.^91^ Sequences that did not align were removed from the dataset and a phylogenetic tree of the filtered aligned sequence data was made using FastTree.^92^ The 16S copy number and V4 primer differences were estimated with the SINAPS algorithm and the UNBIAS reference databases, accessed through USEARCH.^89^

### Quantification and statistical analyses

Analysis of the resulting OTU table was performed in R, utilizing a variety of packages (R Core Team, 2019). The OTU table was converted from BIOM format to PhyloSeq^93^ and normalized by estimated 16S copy number.^94^ The prevalence and abundance of OTUs was determined and OTUs less prevalent or abundant than the first quartile of data were removed from the data set. Relative abundances of OTUs were examined using PhyloSeq.^93^ The OTU table was normalized using Cumulative Sum Scaling, as determined through the R package metagMisc::phyloseq_transform_css. Bray-Curtis dissimilarity values were determined and Principal Coordinate Analysis (PCoA) was performed. Axis 1 and axis 2 were visualized for each plot.

Significant sources of variation were identified using the ‘adonis’ function (permutational ANOVA), run with 1,000 permutations and a Benjamini-Hochberg-Yekutieli false discovery rate correction, available through the Vegan R package. Mouse metadata including parent group, sex, cage, experiment, and genotype were input into the model when available. Significance was defined as p < .01.

The relative abundance of OTUs between experimental groups were compared using MetagenomeSeq, which utilized RNA-seq Limma software adapted for metagenomics,^95^ and DESeq2.^96^ PhyloSeq was used to convert the data to MetagenomeSeq format. Zero-inflated Gaussian (FitZig) function in MetagenomeSeq was primarily used to identify differences between genotypes, retaining results with a p-value <0.01 and a log2 fold change (LFC) > 1. Mouse sex was controlled for and results were filtered to remove species that were not identified in the MetagenomeSeq: EffectiveSampleSize. Results were adjusted for multiple comparisons using the Benjamini-Hochberg-Yekutieli method. Comparisons were achieved using the ‘makeContrasts’ function in MetagenomeSeq. DESeq2 was used to further validate these results. DESeq2 was performed on an OTU table additionally filtered to remove OTUs with less than 10,000 sequences per sample, to reduce the impact of low coverage. The test was performed using a parametric fit and a Wald test, defined by the function nbinomWaldTest, comparing by Genotype*Experiment. OTUs with an absolute LFC > 2 and an adjusted p-value < 0.01 were retained for analysis. These OTUs were used to validate the results from MetagenomeSeq FitZig.

### α-galactosylceramide administration *in* *vivo*

Mice were fasted for 6 h prior to oral gavage with 2 μg αGC in 200 μl PBS or PBS only. In parallel, mice were injected intravenously (i.v.) with 0.5 μg αGC in 100 μl PBS. Spleens and mesenteric lymph nodes (mLN) were analyzed at d3 or d5, as indicated. In some experiments, colons were collected at d3. Proximal colon biopsy punches (2 mm) were cultured in complete RPMI 1640 medium supplemented with 10% FCS for 48 h. Culture supernatants were analyzed using the Quantibody® Mouse Cytokine array from RayBiotech Life, Inc.

### Cell preparation and flow cytometry

Spleens and mesenteric lymph nodes (mLNs) were mechanically disrupted and filtered through 40 µm cell strainers into single cell suspensions. Colons were opened longitudinally, and the mucus scraped off. Small colon sections were shaken in PBS with EDTA (5 mM) and Hepes (5 mM) for 15 min at 37°C, rinsed in 1X HBSS, and gently rubbed on a paper towel to further remove mucus. Tissues were minced using scissors, digested in HBSS buffer containing 4% FBS, 0.5 mg/ml DNAse I, 0.5 mg/ml collagenase IV and 10 mM Hepes at 37°C for 20 min, vortexed vigorously and processed through 40 µm cell strainers. Lymphocytes were isolated through a Percoll density gradient centrifugation, according to the manufacturer specifications. Cells were centrifuged, resuspended in 40% Percoll, and overlaid on 80% Percoll. Recovered cells were resuspended in MACS buffer (0.5 g/L bovine serum albumin and 0.744 g/L ethylenediaminetetraacetic acid) and stained with LIVE/DEAD^TM^ Fixable Aqua and antibodies for surface markers. Cells were then fixed and permeabilized using the Intracellular Fixation & Permeabilization Kit (eBioscience) and stained with antibodies against transcription factors for 30 min. iNKT cells were identified as live CD19^−^ TCRβ^+^ PBS57-loaded CD1d Tetramer^+^ lymphocytes. iNKT cell subsets were discriminated by expression of the transcription factors PLZF, T-bet and RORγt, as previously described.^51^ Samples were acquired on the LSR Fortessa cytometer in the Temerty Faculty of Medicine Flow Cytometry Facility at the University of Toronto. FlowJo (BD Biosciences) was used to analyze flow cytometry data and GraphPad Prism was used for the Mann-Whitney and *t* tests.

## Supplementary Material

Supplemental Material

## Data Availability

All the sequencing data generated in this manuscript is available at the NCBI Sequence Read Archive (SRA) at https://www.ncbi.nlm.nih.gov/sra/PRJNA692215 under the BioProject ID PRJNA692215.
