# Supplementary material for: Invariant natural killer T cells minimally influence gut microbiota composition in mice
Source: Gut Microbes. 2022 Jul 31;14(1):2104087. doi: 10.1080/19490976.2022.2104087 (PMC9348128; doi:10.1080/19490976.2022.2104087)
Supplement: Supplemental Material [file KGMI_A_2104087_SM7624.zip › Supplementary Material Revised.docx]

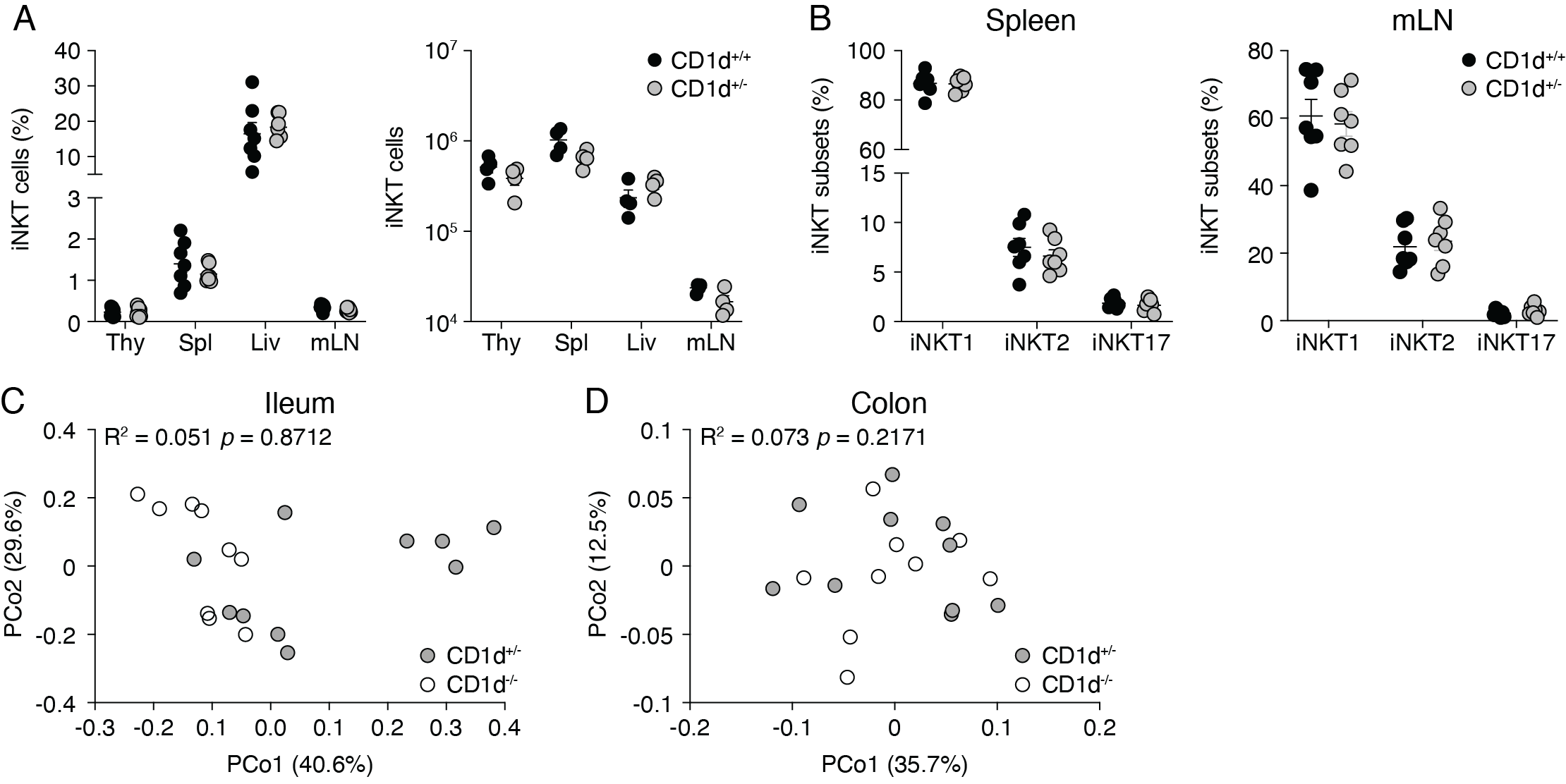


**Supplementary Figure 1.** **CD1d-deficiency does not alter microbiota composition.** (**A**) Frequency (left panel) and absolute number (right panel) of TCRβ^+^ PBS57-CD1d tetramer-positive iNKT cells out of live CD19^-^ lymphocytes in the indicated tissues. (**B**) Frequency of iNKT cell subsets in the spleen and mesenteric lymph nodes (mLN). Data shows individual and mean values +/- s.e.m. (n = 4 to 7 mice per group). (**C**, **D**)16S bacterial rRNA sequencing was used to define the microbiota profiles from the ileum (C) and colon (D) of CD1d^+/-^ (n = 10) and CD1d^-/-^ (n = 9) littermate mice. These groups were separated by principal coordinates PCo1 and PCo2, based on Bray-Curtis distances. Data shows R^2^ (effect size) and adjusted *p* values for the genotype.


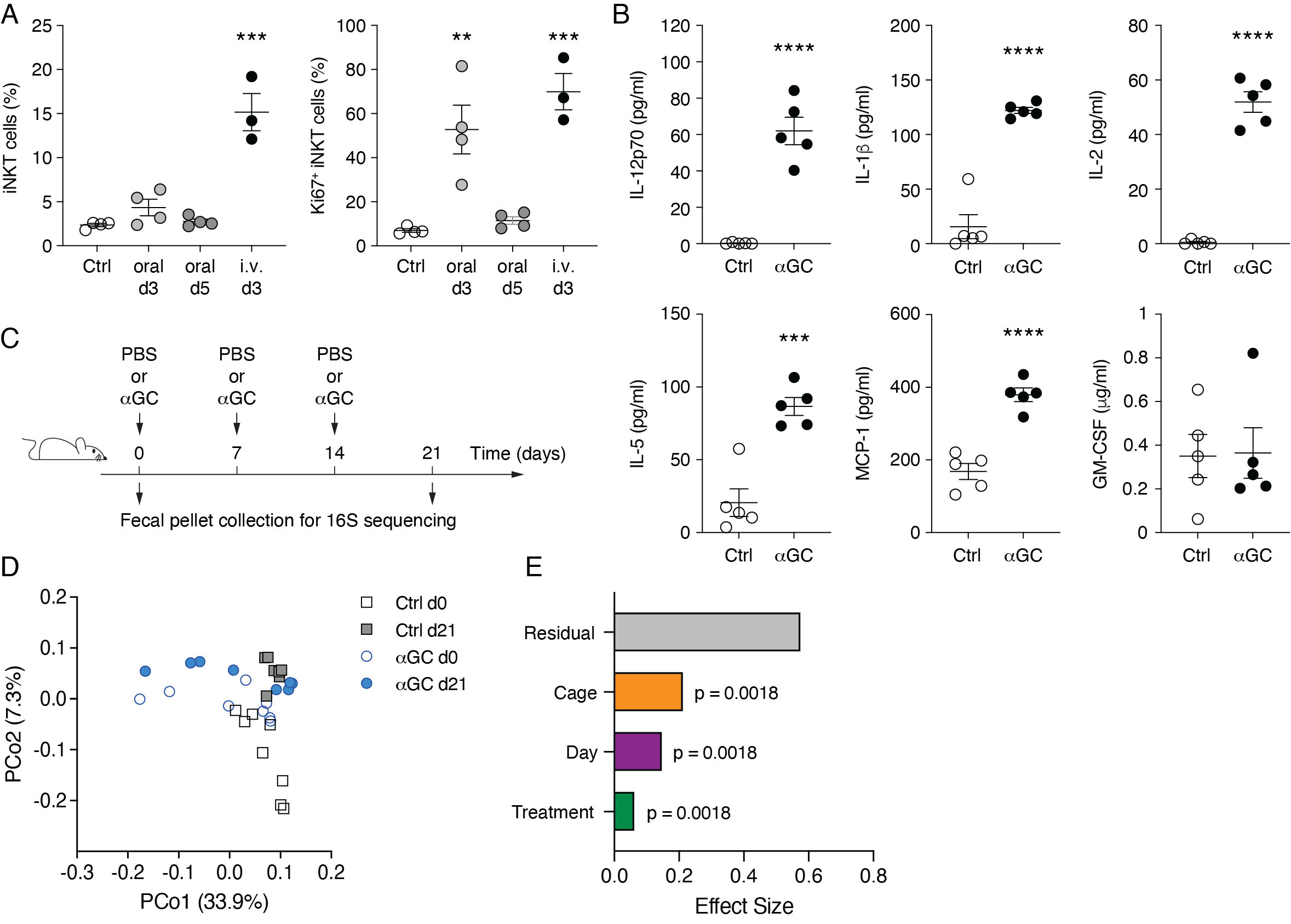


**Supplementary Figure 2.** **Repeated** **iNKT cell activation does not affect microbiota composition.** (**A**) C57BL/6 mice were administered 2 μg αGC or vehicle control (Ctrl) orally, or 0.5 μg αGC intravenously (i.v.) and their spleens were analyzed at d3 and d5 (for oral) and d3 (i.v.). (**B**) C57BL/6 mice were administered 2 μg αGC or vehicle control (Ctrl). At d3, colon biopsy punches were cultured for 48 h and cytokines production was assessed using a multiplex cytokine array. Data shows individual and mean values +/- s.e.m. (n = 3 to 5 mice per group). **p* < 0.05, ***p* < 0.01, *** *p* < 0.001, Unpaired Student *t* test (A-C). (**C)** Schematic of the repeated αGC administration procedure. (**D**) 16S bacterial rRNA sequencing was used to define the microbiota profiles of mice before (d0) and after (d21) after the repeated oral gavage of αGC or vehicle control (PBS). These groups were separated by principal coordinates PCo1 and PCo2, collectively explaining 41.2% of the total similarity between samples, based on Bray-Curtis distances. (**E**) Permutational multivariate analysis of the variance using Adonis. Data shows R^2^ (effect size) and adjusted *p* values for treatment (αGC vs. PBS), day (d0 vs. d21) and caging.
